# Role for DNA Methylation in the Regulation of miR-200c and miR-141 Expression in Normal and Cancer Cells

**DOI:** 10.1371/journal.pone.0008697

**Published:** 2010-01-13

**Authors:** Lukas Vrba, Taylor J. Jensen, James C. Garbe, Ronald L. Heimark, Anne E. Cress, Sally Dickinson, Martha R. Stampfer, Bernard W. Futscher

**Affiliations:** 1 Arizona Cancer Center, The University of Arizona, Tucson, Arizona, United States of America; 2 Department of Pharmacology & Toxicology, College of Pharmacy, The University of Arizona, Tucson, Arizona, United States of America; 3 Life Sciences Division, Lawrence Berkeley National Laboratory, Berkeley, California, United States of America; 4 Biology Centre ASCR, v.v.i., Institute of Plant Molecular Biology, Ceske Budejovice, Czech Republic; Victor Chang Cardiac Research Institute, Australia

## Abstract

**Background:**

The microRNA-200 family participates in the maintenance of an epithelial phenotype and loss of its expression can result in epithelial to mesenchymal transition (EMT). Furthermore, the loss of expression of miR-200 family members is linked to an aggressive cancer phenotype. Regulation of the miR-200 family expression in normal and cancer cells is not fully understood.

**Methodology/Principal Findings:**

Epigenetic mechanisms participate in the control of miR-200c and miR-141 expression in both normal and cancer cells. A CpG island near the predicted mir-200c/mir-141 transcription start site shows a striking correlation between miR-200c and miR-141 expression and DNA methylation in both normal and cancer cells, as determined by MassARRAY technology. The CpG island is unmethylated in human miR-200/miR-141 expressing epithelial cells and in miR-200c/miR-141 positive tumor cells. The CpG island is heavily methylated in human miR-200c/miR-141 negative fibroblasts and miR-200c/miR-141 negative tumor cells. Mouse cells show a similar inverse correlation between DNA methylation and miR-200c expression. Enrichment of permissive histone modifications, H3 acetylation and H3K4 trimethylation, is seen in normal miR-200c/miR-141-positive epithelial cells, as determined by chromatin immunoprecipitation coupled to real-time PCR. In contrast, repressive H3K9 dimethylation marks are present in normal miR-200c/miR-141-negative fibroblasts and miR-200c/miR-141 negative cancer cells and the permissive histone modifications are absent. The epigenetic modifier drug, 5-aza-2′-deoxycytidine, reactivates miR-200c/miR-141 expression showing that epigenetic mechanisms play a functional role in their transcriptional control.

**Conclusions/Significance:**

We report that DNA methylation plays a role in the normal cell type-specific expression of miR-200c and miR-141 and this role appears evolutionarily conserved, since similar results were obtained in mouse. Aberrant DNA methylation of the miR-200c/141 CpG island is closely linked to their inappropriate silencing in cancer cells. Since the miR-200c cluster plays a significant role in EMT, our results suggest an important role for DNA methylation in the control of phenotypic conversions in normal cells.

## Introduction

miRNAs are single-stranded, 20-24 nt long RNAs that regulate gene expression at the posttranscriptional level. miRNAs frequently target 3′ UTRs of mRNA, and since miRNA target motifs do not require complete homology, hundreds of mRNA targets may exist for each miRNA. Current estimates are that there are nearly 900 unique miRNAs encoded in the human genome, and these miRNAs control, in part, the expression of more than one third of human genes [1]. A number of miRNA dysregulated in human cancer have been shown to have oncogenic or tumor suppressive activity [2], [3], [4]. These include miRNA species that show cell type specific patterns of expression, some of which are important in the maintenance of cell identity [5], [6]. These types of miRNA are prime targets for epigenetic control, and early studies of miRNA control support this possibility [7], [8].

miR-200c and miR-141 are members of the miR-200 family and are important regulators of the epithelial to mesenchymal transition (EMT) [5], [9], [10], [11]. In addition to the role of miR-200c and miR-141 in the phenotypic conversion of normal cells, dysregulation of normal patterns of miR-200c expression occurs in multiple types of cancer cells and is linked to tumor progression [2], [6], [12], [13], [14], [15]. The mechanism responsible for the control of miR-200c expression in both normal and cancer cells is not fully understood. In this study, we show that the epigenetic state is closely linked to normal cell type specific expression of miR-200c and miR-141, and this epigenetic state is dysregulated in carcinoma cells, where loss of miR200c/141 expression is linked to aberrant DNA methylation and histone modifications. Finally, we found that the miR-200c regulation by DNA methylation is evolutionarily conserved between humans and mice. Since miR-200c plays a significant role in EMT, our results suggest that DNA methylation plays an important role in the control of phenotypic conversions of normal and cancer cells.

## Results and Discussion

**Figure 1:**
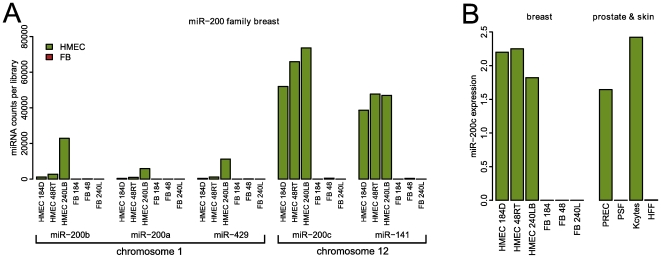
miR-200c is expressed in an epithelial selective fashion. A. miR-200 family expression according to massive parallel sequencing of small RNA libraries from a set of three isogenic pairs of human mammary epithelial cells (HMEC) and fibroblasts (FB). The expression of the mir-200b-200a-429 cluster located on human chromosome 1, and the expression of the mir-200c-141 cluster located on human chromosome 12 are shown. With the average of 63,829 counts out of 3,926,984 per library in HMEC, miR-200c forms 1.625% of all small RNAs in these cells. B. Real-time PCR assessment of miR-200c expression in normal cell types. The left panel shows the expression of miR-200c in the same samples as panel A. The right panel shows the expression of miR-200c in human prostate epithelial cells (PREC), prostate stromal fibroblasts (PSF), human skin keratinocytes (Kcytes) and skin fibroblasts (HFF). The data are normalized relative to let-7a, which is expressed at equivalent levels between different samples according to the small RNA sequencing data. Real time PCR analysis of miR-141 expression in these samples is provided in Figure S1.

The miR-200 family is comprised of five miRNAs that are encoded within two clusters. Each cluster encodes a polycistronic gene. One cluster resides on human chromosome 1 and encodes miR-200b, miR-200a, and miR-429, while the other cluster is located on human chromosome 12, and encodes miR-200c and miR-141. Our small RNA library sequencing data (Figure 1A) show that the miR-200 family is highly expressed in cultured normal human mammary epithelial cells (HMEC) derived from three different individuals, whereas the isogenic human mammary fibroblast cells (FB) lack miR-200 family expression (Figure 1A). It is apparent from the small RNA library sequencing data (Figure 1A) that the most highly expressed members of the miR-200 family in HMEC are miR-200c and miR-141. We corroborated the expression of miR-200c and miR-141 in the same set of normal mammary samples by real-time PCR, and then expanded these results to pairs of epithelial cells and fibroblasts from prostate and skin, as well (Figure 1B; Figure S1). In all cases, miR-200c and miR-141 were highly expressed in epithelial cells, but were not expressed in fibroblasts.

**Figure 2:**
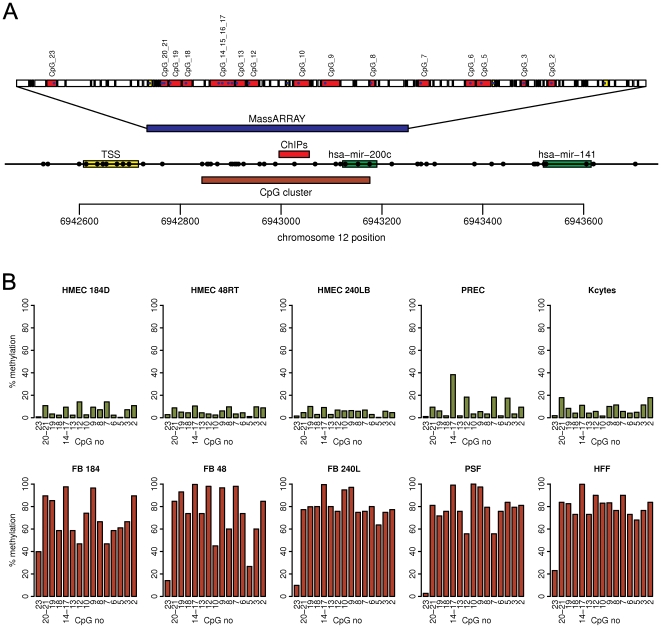
The mir-200c CpG island shows differential cytosine methylation between miR-200c-positive and miR-200c-negative normal human tissues. A. A diagram of the genomic region of hsa-mir-200c. The top bar shows the specific fragments analyzed by MassARRAY. The red fragments named with CpG sites indicate the fragments from which DNA methylation data was obtained. This data is presented panel B. Below this is the region analyzed by MassARRAY in relation to the genomic location (in blue), followed by the region of the real-time PCR amplicon for chromatin immunoprecipitation analysis (ChIP), and the CpG island identified by the program CpGcluster. The regions encoding the mir-200c and mir-141 hairpins and the putative transcription start (TSS) region inferred from the human EST track of the UCSC genome browser are displayed, and each circle on this track represents the position of a CpG dinucleotide. The ruler at the bottom shows the location on human chromosome 12 according to human genome assembly hg18. B. Summary of 5-methylcytosine levels obtained by MassARRAY analysis of the hsa-mir-200c CpG island in samples characterized in Figure 1B. The y-axis shows the percent of cytosine methylation within the individual CpG units marked on x-axis. The CpG units within the MassARRAY amplicon are numbered in the reverse direction, with CpG 2 being located within the miR-200c coding sequence.

The mir-200c hairpin coding sequence and approximately 300 bp of upstream genomic sequence is CpG rich. According to our calculations using the program CpG Cluster [16] this region is a highly statistically significant CpG cluster (Figure 2A). This CpG cluster (length 334 bp, GC% 68.56, O/E ratio 0.58, 21CpGs, p-value 8.44×10^−11^) has the characteristics close to a CpG island definition based on size, GC content and CpG dinucleotide frequency [17], as well as its location with respect to the transcriptional unit [18]. In addition, this region is considered a CpG island based on a recently published probabilistic definition [19]. We analyzed this region as a possible target of epigenetic control.

The epigenetic state of the miR-200c/141 CpG island shows clear and extensive cell type specific differences between normal miR-200c/141-positive and miR-200c/141-negative cells. We used MassARRAY technology to analyze the DNA methylation state of the mir-200c cluster CpG island (Figure 2B). Results show that the CpG sites are unmethylated in three separate strains of miR-200c/miR-141-positive HMEC. In contrast, all the CpG sites are highly methylated in the isogenic miR-200c/miR-141-negative fibroblast strains. The inverse correlation between miRNA expression and DNA methylation extends to other miR-200c/miR-141-positive/negative pairs of normal cells, such as prostate epithelial cells and skin keratinocytes, and their mesenchymal cell type counterparts, prostate and skin fibroblasts. Thus, the miR-200c cluster CpG island is unmethylated in normal miR-200c/miR-141-positive epithelial cells, while being densely methylated in the paired normal miR-200c/miR-141-negative fibroblasts (Figure 1B, Figure 2B, Figure S2).

**Figure 3:**
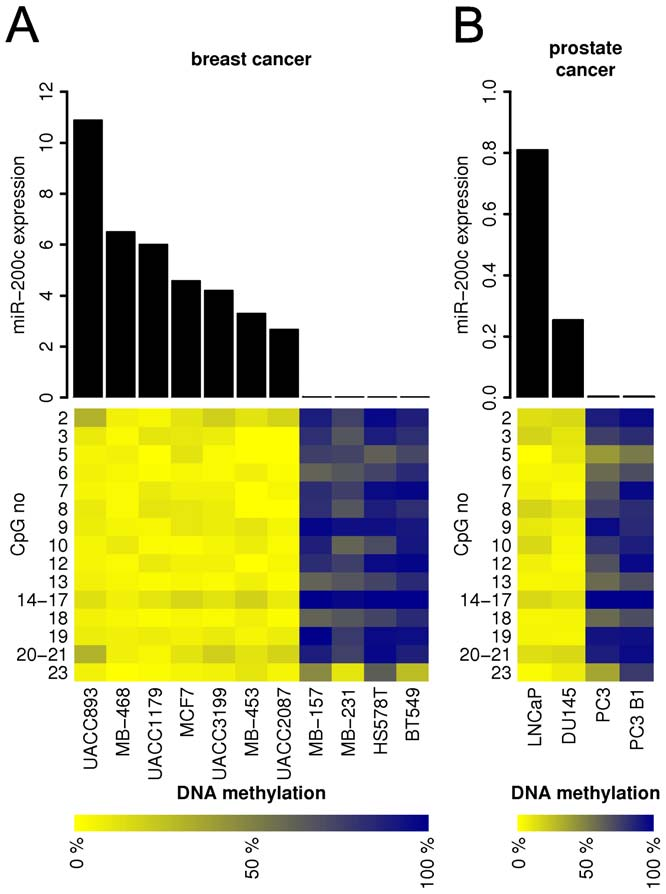
DNA methylation of mir-200c CpG island in breast and prostate cancer cell lines. **A.** miR-200c expression and mir-200c CpG island methylation in eleven breast cancer cell lines. **B.** miR-200c expression and mir-200c CpG island methylation in four prostate cancer cell lines. The top panel of each figure shows the expression of miR-200c in cancer samples as detected by real-time PCR, normalized to let-7a. The bottom panel shows the methylation level of the mir-200c CpG island region in the same cancer samples. The level of methylation of individual CpG units within the MassARRAY amplicon is displayed as a heatmap with the lowest methylation in yellow and the highest methylation in blue. The y-axis marks the individual CpG units.

miR-200c and miR-141 expression is lost in different types of cancer cells [5], [11], [20], [21], and we sought to determine if this loss of expression was linked to epigenetic changes in the miR-200c/miR-141 CpG island. We analyzed 11 breast cancer cell lines, and in each case, miR-200c and miR-141 expression was closely linked to the DNA methylation state of the CpG island (Figure 3A; Figure S3). Seven of the breast cancer cell lines tested express miR-200c and miR-141 and each has an unmethylated mir-200c CpG island. The other four breast cancer cell lines tested do not express miR-200c and miR-141 and exhibit a densely methylated mir-200c CpG island. A similar picture emerges with respect to prostate cancer cells. We show two prostate cancer cell lines (PC3 and PC3 B1) where loss of miR-200c and miR-141 expression is linked with aberrant DNA methylation of the mir-200c/141 CpG island (Figure 3B; Figure S3), and two prostate cancer cell lines (LNCaP and DU145) that retain miR-200c/miR-141 expression and an unmethylated mir-200c/141 CpG island. Together these results indicate that cancer cells derived from normal miR-200c/miR-141-positive epithelial cells can replicate the cell type-specific DNA methylation pattern of the miR-200c/141 CpG island seen in normal miR-200c/miR-141-negative cells, and that the aberrant DNA methylation of the miR200c/141 CpG island in these cancer cells is associated with its transcriptional silencing in carcinoma cells.

**Figure 4:**
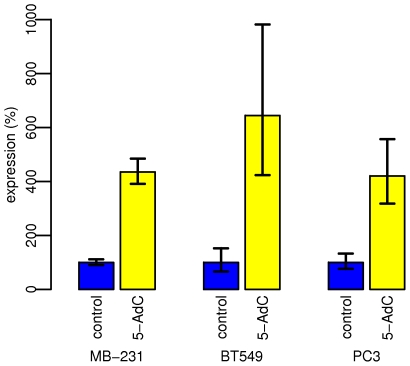
5-aza-2′-deoxycytidine treatment reactivates miR-200c expression in breast and prostate cancer cell lines. Cells were treated with 3 µM 5-aza-2′-deoxycytidine for 96 h. The level of expression of miR-200c was measured by real-time PCR. The average of 4 independent samples is displayed, the error bars show the standard error of measurement. The values were normalized to untreated controls (100%). Figure S4 shows the 5-aza-2′-deoxycytidine-mediated reactivation of miR-141 in the same samples.

To demonstrate the functional significance of the epigenetic state of the miR-200c/mir-141 CpG island in cancer cells, we exposed cancer cells to the epigenetic modifier and DNA methyltransferase inhibitor 5-aza-2′-deoxycytidine (5-AdC). The miR-200c/miR-141-negative breast cancer cell lines MDA-MB-231 and BT549 and prostate cancer cell line PC3 were treated with 3 µM 5-AdC for 96 h and miR-200c/141 expression was assessed by real-time PCR. Figure 4 shows 5-AdC reactivated miR-200c expression in all three cancer cell lines. The level of miR-200c increased 4.3-fold in MDA-MB-231 (p-value = 0.0004), 6.4-fold in BT549 (p-value = 0.0107) and 4.2-fold in PC3 cells (p-value = 0.0072). A similar reactivation of miR-141 expression (p-value<0.01) was also observed in these cancer cell lines after 5-AdC treatment (Figure S4). These data suggest that epigenetic mechanisms participate in the inappropriate repression of miR-200c/miR-141 expression in cancer cells.

**Figure 5:**
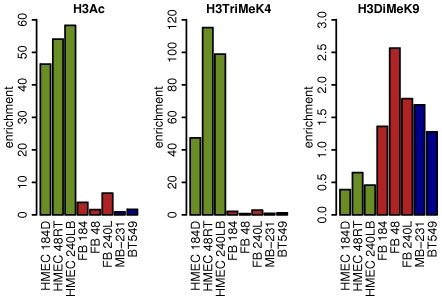
The histone modification state of the mir-200c CpG island. Permissive histone marks represented by acetylation of histone H3 (H3Ac) and trimethylation of lysine 4 of histone H3 (H3TriMeK4) as well as the repressive histone mark dimethylation of lysine 9 of histone H3 (H3diMeK9) were analyzed using chromatin immunoprecipitation coupled to real-time PCR of the region described in Figure 2A. HMEC samples are shown in green, isogenic FB samples are shown in red, and two miR-200-negative breast cancer cell lines are in blue. The y-axis shows fold enrichment of each histone mark over input DNA within the mir-200c CpG island.

The histone modification state of the mir-200c cluster CpG island also shows cell type-specific differences that are closely linked to the expression state of miR-200c/141 in normal and cancer cells. Figure 5 shows the results of chromatin immunoprecipitations coupled to quantitative real-time PCR analysis that were used to examine the histone modification state of the miR-200c/141 CpG island in normal and cancer cells. The CpG island of mir-200c/141 in the three different strains of miR-200c/miR-141-positive HMEC exists in a transcriptionally competent state; it is enriched for the transcriptionally permissive modifications of histone H3 acetylation (H3Ac) and lysine 4 trimethylation (H3TriMeK4), while the transcriptionally repressive histone mark of histone H3 lysine 9 dimethylation (H3DiMeK9) is underrepresented (Figure 5). In contrast, in the isogenic miR-200c/miR-141-negative mammary fibroblasts permissive histone modifications are absent, and the repressive H3 lysine 9 dimethylation mark is present (Figure 5). Similarly, the breast cancer cell lines that had lost miR-200c/141 expression lost histone H3 acetylation and K4 trimethylation and acquired a repressive histone state, enriched for the H3 lysine 9 dimethylation mark (Figure 5). No enrichment of trimethylation of histone H3 lysine 27 was detected in the miR-200c/141 CpG island in the samples analyzed (Figure S5). Taken together, the results from the analyses of miR-200c/141 expression, DNA methylation, and histone modification states across a variety of normal and cancer cell types demonstrate a close link between the expression of mir-200c/141 and the epigenetic state of their associated CpG island.

**Figure 6:**
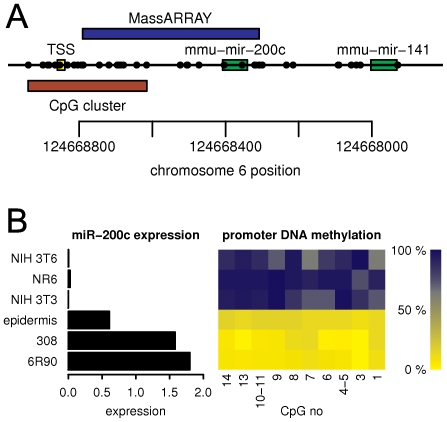
Epigenetic control of miR-200c expression is evolutionarily conserved. **A.** Diagram of the mouse mmu-mir-200c genomic interval. The top bar shows the area analyzed by MassARRAY. The regions encoding the hairpins of mir-200c and mir-141 and the putative transcription start (TSS) inferred from the mouse EST track displayed on the UCSC genome browser are shown, and each circle on this track represents the location of a CpG dinucleotide. Similar to the human hsa-mir-200c, the mouse mmu-mir-200c contains a CpG island, identified by the program CpG Cluster. The ruler at the bottom shows the location on mouse chromosome 6 according to genome assembly mm9. The genes are encoded on the (-) strand. **B.** Mouse cells show a similar cell type specific pattern in miR-200c expression to human cells and this expression is linked to the DNA methylation state of the CpG island. The left panel shows the expression of miR-200c in mouse epithelial cells (308, 6R90, SKH-1 epidermis) and mouse fibroblast cell lines (NIH 3T3, NR6, NIH 3T6) as detected by real-time PCR. The right panel shows the methylation level of the mir-200c CpG island region in the same mouse samples. The level of methylation of individual CpG units within the MassARRAY amplicon is displayed as a heatmap with the lowest methylation in yellow and the highest methylation in blue. The x-axis marks the individual CpG units. CpG units within MassARRAY amplicon are numbered in reverse direction, with CpG 1 being located within the miR-200c coding sequence.

Finally, we sought to determine if the epigenetic regulation of miR-200c expression in normal cells is conserved evolutionarily, reasoning that DNA methylation-linked control of miR-200c expression across mammalian species would provide further experimental support for epigenetic control of cell-type specific expression of miR-200c. The whole genomic cluster containing mir-200c and mir-141 is well conserved between the human and mouse genome. Similar to the human mir-200c/141 genomic region, the mouse mir-200c/141 genomic region also contains a CpG island (Figure 6A; length 325 bp, GC% 66.77, O/E ratio 0.58, 19 CpGs, p-value 1.06×10^−10^). To evaluate a potential role for DNA methylation in the control of miR-200c/141 in mice, CpG methylation and miRNA expression were analyzed in mouse epithelial cells (epidermis of SKH-1 mouse and keratinocyte cell lines 308 and 6R90) and mouse fibroblasts (cell lines NIH 3T3, NIH 3T6, and NR6). A MassARRAY amplicon was designed to analyze the DNA methylation state of the mouse mir-200c/141 region homologous to that analyzed in human (Figure 6A). Strikingly similar results for miR-200c were found between the human cells and mouse cells. Mouse keratinocytes expressed significant levels of miR-200c, while the mouse fibroblasts did not express detectable levels of miR-200c (Figure 6B). DNA methylation analysis by MassARRAY revealed that the miR-200c-positive keratinocytes showed minimal DNA methylation in the mir-200c CpG island, while the miR-200c-negative mouse fibroblasts showed extensive DNA methylation of all CpG sites in the region (Figure 6B). The significant conservation in DNA sequence, patterns of cell type-specific DNA methylation, and the associated miR-200c expression patterns between the human and mouse genomes, which are separated by 75 million years of evolution [22], provides evidence that epigenetic mechanisms play a functional role in the control of miR-200c expression.

In summary, our findings provide multiple lines of evidence that epigenetic mechanisms are involved in the regulation of miR-200c/141 expression in both normal and cancer cells. First, there is a consistent inverse correlation between expression and DNA methylation states in normal human and mouse cell types, as well as human breast and prostate cancer cell lines. Second, different histone codes exist between miR-200c/141 expressing and non-expressing cells that accurately mirror the expression and DNA methylation states. Third, the epigenetic modifier 5-aza-2′-deoxycytidine relieves the repression of miR-200c/miR-141 in cancer cell lines. Fourth, the link between DNA methylation and expression states occurs across mammalian species, since it is seen in human and mouse. Taken together, these findings indicate that miR-200c/141 is an evolutionarily conserved epigenetically labile miRNA cluster.

Dysregulation of miR-200c and miR-141 occurs in multiple cancer types [5], [11], [20], [21], [23], [24], [25], [26], and this dysregulation involves a compromise of the epigenetic state of the CpG island associated with miR-200c and miR141. Results suggest that these carcinoma cells may co-opt *de novo* DNA methylation pathways involved in the epigenetic control of normal cell type-specific genes, such as those that govern the epigenetic state of miR-200c/miR-141. A similar apparent co-option of cell type specific DNA methylation pathways by cancer cells is also seen in protein-coding genes, such as *maspin* and *14-3-3 sigma* [27], [28]. Together these results suggest that pathways responsible for the establishment or maintenance of normal cell type-specific DNA methylation states may be disrupted during carcinogenesis.

Since miR-200c and miR141 play an important role in EMT and therefore cell identity, disruption of mechanisms that govern cell type specific DNA methylation patterns during carcinogenesis could likely effect expression of miR-200c and miR141 and provide phenotypic plasticity to cancer cells. Support of this possibility comes from the phenotypes of the cancer cells analyzed in this study. All four of the breast cancer cell lines that lost miR-200c and miR-141 expression have an aberrantly methylated mir-200c/141 CpG island, and each of these cell lines displays a mesenchymal phenotype [11], [29]. In contrast, those breast cancer cell lines that express miR-200c and miR-141 and have an unmethylated CpG island display an epithelial phenotype [11], [29]. A similar picture emerges in the prostate cancer cell lines. The PC3 cells that have lost miR-200c and miR-141 expression, display an aberrantly methylated CpG island and a mesenchymal phenotype, whereas LnCaP and Du145 retain miR-200c and miR-141 expression and an epithelial phenotype [11], [30]. These results suggest that DNA methylation may control the phenotypic changes observed in cancer cells.

## Materials and Methods

### Cell lines and cell culture

Finite lifespan pre-stasis HMEC from specimens 184 (batch D), 48R (batch T), and 240L (batch B), were derived from reduction mammoplasty tissue of women aged 21, 16, and 19 respectively. Cells were initiated as organoids in primary culture in serum-containing M85 medium supplemented with oxytocin (Bachem) at 0.1 nM, and maintained in M87A medium supplemented with oxytocin and cholera toxin at 0.5 ng/ml [31]. Fibroblasts from specimens 184, 48, and 240 L were obtained from the same reduction mammoplasty tissue and were grown in DMEM/F12 with 10% FBS and 10 µg/ml insulin [31] and further propagated in DMEM/F12 with 10% FBS. Prostate epithelial cells were obtained from Clonetics (San Diego, CA), and fetal skin keratinocytes from Cell Applications (San Diego, CA.) and were grown according to the suppliers instructions. Human foreskin fibroblasts (HFFs) were maintained and cultured by the Arizona Cancer Center Cell Culture Shared Service. Human prostate stromal fibroblasts (PSF) were were cultured as previously described [32]. Breast cancer cell lines BT549, HS578T, MCF7, MDA-MB-157, MDA-MB-231, MDA-MB-453, MDA-MB-468, UACC893, UACC1179, UACC2087, and UACC3199 were cultured as previously described [33], [34]. Prostate cancer cell lines PC3, PC3 B1, LNCaP, and DU145 [35], [36], [37] were maintained in RPMI 1640 medium containing 10% fetal bovine serum supplemented with 100 units/ml penicillin and 50 µg/ml streptomycin. Mouse keratinocyte cell lines 308 and 6R90 were cultured as described [38], mouse fibroblast cell lines NIH 3T3, NIH 3T6 and NR6 [39], [40], [41] were maintained in DMEM medium containing 10% fetal bovine serum. SKH-1 mouse epidermis samples were removed from liquid nitrogen snap frozen dorsal skin by scraping on dry ice.

miRNA library preparation, sequencing and analysis

Total RNA was extracted using Trizol. The small RNA fraction was purified on a 15% polyacrylamide-urea gel. A preadenylated adaptor was ligated to the 3′ end of the small RNA followed by purification of the ligation product on a 15% PAA-urea gel. An Illumina specific 5′ adaptor was ligated and the product was purified on a 10% PAA-urea gel. Small RNA with ligated adaptors was reverse transcribed into DNA using a RT primer with Illumina specific extension. cDNA was then PCR amplified using Illumina specific primers and the PCR product was purified on a 3% agarose gel. Small RNA libraries were submitted for Illumina sequencing to NCGR (Santa Fe, NM). Reads from Illumina GAII were mapped to the hg18 human genome assembly using program Novoalign (www.novocraft.com). Output from Novoalign was further analyzed in R (http://www.r-project.org). The counts of individual miRNAs were normalized for average library size (3,926,984 counts).

### Nucleic acid isolation

RNA was isolated using either Trizol (Invitrogen) or the RNeasy Mini kit (Qiagen) and quantified by absorption measurements at 260 nm. Genomic DNA was isolated using the DNeasy Blood and Tissue Kit (Qiagen) and quantified spectrophotometrically.

### Real-time PCR detection of miRNA

Real-time PCR detection of microRNAs was performed in principle as described [42]. Reverse transcription was performed using TaqMan Reverse Transcription Reagents (Applied Biosystems, Foster City, CA, USA). Real-time PCR was conducted on an ABI Prism 7500 Sequence Detection System (Applied Biosystems, Foster City, CA, USA) using PerfeCta SYBR Green SuperMix, Low ROX (Quanta Biosciences, Gaithersburg, MD, USA) with a 95°C denaturation for 3 minutes followed by 40 cycles of 95°C for 15 seconds and 60°C for 45 seconds. Differences in expression were determined using the comparative Ct method described in the ABI user manual relative to let-7a. Primer sequences are listed in Table S1.

### CpG island prediction

CpG islands were predicted using the program CpGcluster [16]. This program uses a statistical approach to search for regions with significant enrichment of CpG dinucleotides rather than parameters within a sliding window. We set the threshold to 50 (median distance) and p-value cut to 10^−8^.

### DNA methylation analysis by MassARRAY

DNA methylation analysis by MassARRAY was performed as described [43]. Primer sequences are listed in Table S1.

### 5-aza-2′-deoxycytidine treatment

Cells were treated with 3 µM 5-aza-2′-deoxycytidine (Sigma, St Louis, MO, USA) for 96 h, as previously described [44].

### Chromatin immunoprecipitation

Chromatin immoprecipitation (ChIP) analysis was performed as described previously [33], [45], [46] with antibodies against acetylated histone H3 (#06-599, Millipore), trimethylated histone H3 K4 (#05-745, Upstate), dimethylated histone H3 K9 (CS200587, Millipore), and trimethylated histone H3 K27 (#07-449, Millipore). Equal amounts (1 ng) of ChIP and input DNA were used for real-time PCR analysis. Primers were designed for use with the Human Universal Probe Library Set (Roche Diagnostics, Indianapolis, IN, USA). Real-time PCR was conducted on an ABI Prism 7500 Sequence Detection System (Applied Biosystems, Foster City, CA, USA) using PerfeCta qPCR SuperMix, Low ROX (Quanta Biosciences, Gaithersburg, MD, USA) with a 95°C denaturation for 3 minutes followed by 40 cycles of 95°C for 15 seconds and 60°C for 45 seconds. Primer sequences are listed in Table S1.

## Supporting Information

Figure S1 Real-time PCR assessment of miR-141 expression in normal cell types. The left panel shows the expression of miR-141 in three isogenic pairs of mammary epithelial cells (HMEC) and mammary fibroblasts (FB). The right panel shows the expression of miR-141 in human prostate epithelial cells (PREC), prostate stromal fibroblasts (PSF), human skin keratinocytes (Kcytes) and skin fibroblasts (HFF). The data are normalized relative to let-7a, which is expressed at consistent levels between different samples according to the small RNA sequencing data.
(0.13 MB TIF)

Figure S2 DNA methylation of the mir-200c CpG island inversely correlates with miR-200c expression in normal human samples. This figure summarizes data shown in Figure 1B and 2B. The upper panel shows the expression of miR-200c detected by real-time PCR. The bottom panel shows the methylation level of mir-200c CpG island region in the same human samples. The level of methylation of individual CpG units within the MassARRAY amplicon is displayed as a heatmap with the lowest methylation in yellow and the highest methylation in blue. The y-axis marks the individual CpG units.
(0.19 MB TIF)

Figure S3 Real-time PCR assessment of miR-141 expression in breast and prostate cancer cell lines. The left panel shows the expression of miR-141 in eleven human breast cancer cell lines. The right panel shows the expression of miR-141 in four human prostate cancer cell lines.
(0.14 MB TIF)

Figure S4 miR-141 expression in cancer cell lines is reactivated by 5-aza-2′-deoxycytidine treatment. Cells were treated with 3 µM 5-AdC for 96 h. The level of expression of miR-141 was measured by real-time PCR. The average of 4 measurements is displayed, the error bars show the standard error of measurement. The values were normalized to untreated controls (100%).
(0.06 MB TIF)

Figure S5 Histone H3 K27 trimethylation state of the mir-200c CpG island. Histone H3 lysine 27 trimethylation levels of the region of the mir-200c CpG island described in Figure 2A were analyzed by chromatin immunoprecipitation coupled to real-time PCR. Epithelial cells (HMEC) are shown in green and their isogenic fibroblasts (FB) are shown in red. The y-axis shows a lack of enrichment of the histone H3 K27 trimethylation mark within the mir-200c CpG island relative to input DNA in all the samples analyzed.
(0.08 MB TIF)

Table S1 List of primer sequences used in the study
(0.03 MB PDF)
