# Supplementary figures and images for: Role for DNA Methylation in the Regulation of miR-200c and miR-141 Expression in Normal and Cancer Cells
Source: PLoS One. 2010 Jan 13;5(1):e8697. doi: 10.1371/journal.pone.0008697 (PMC2805718; doi:10.1371/journal.pone.0008697)

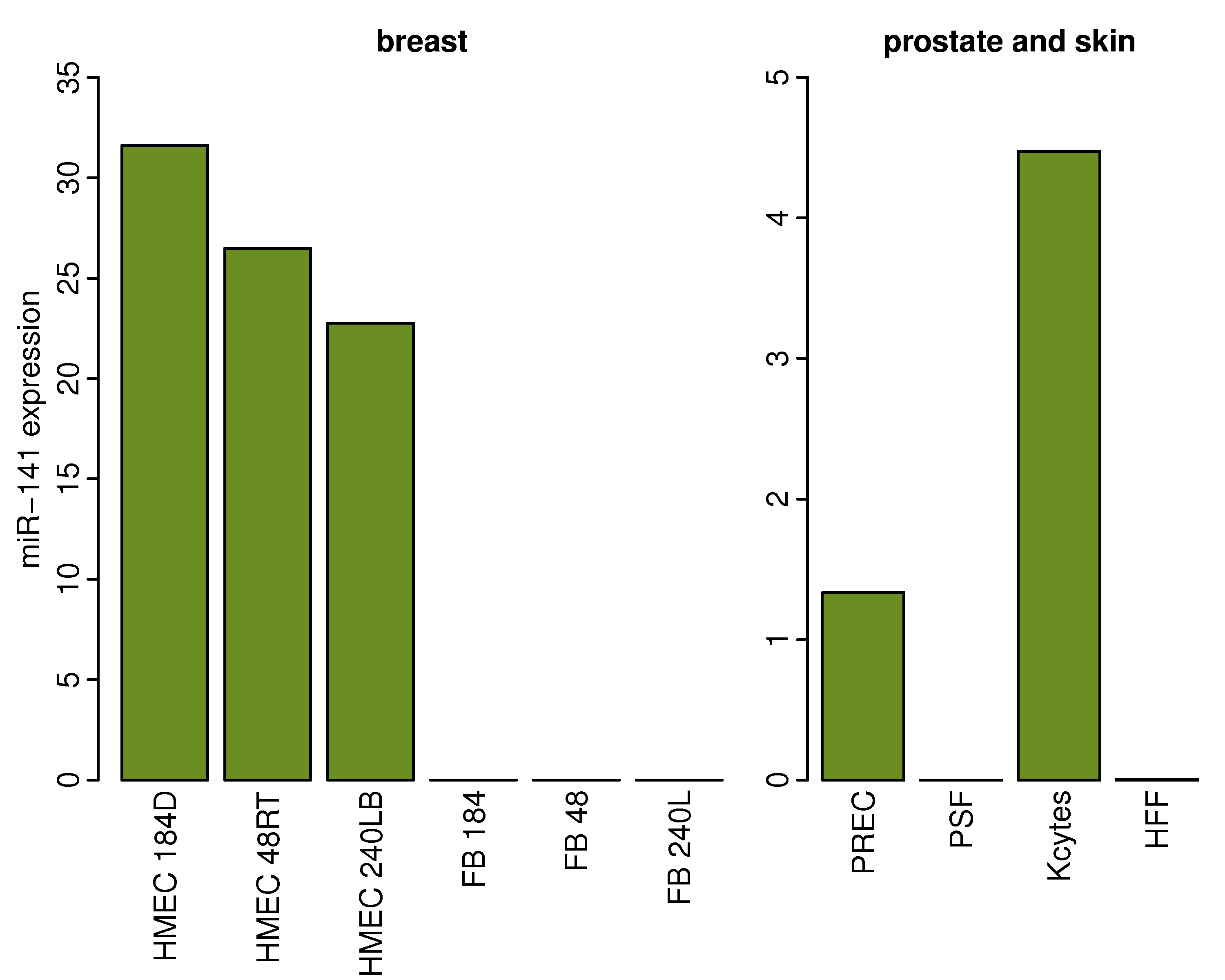

Supplement: Figure S1 — Real-time PCR assessment of miR-141 expression in normal cell types. The left panel shows the expression of miR-141 in three isogenic pairs of mammary epithelial cells (HMEC) and mammary fibroblasts (FB). The right panel shows the expression of miR-141 in human prostate epithelial cells (PREC), prostate stromal fibroblasts (PSF), human skin keratinocytes (Kcytes) and skin fibroblasts (HFF). The data are normalized relative to let-7a, which is expressed at consistent levels between different samples according to the small RNA sequencing data. (0.13 MB TIF) [file pone.0008697.s001.tif]

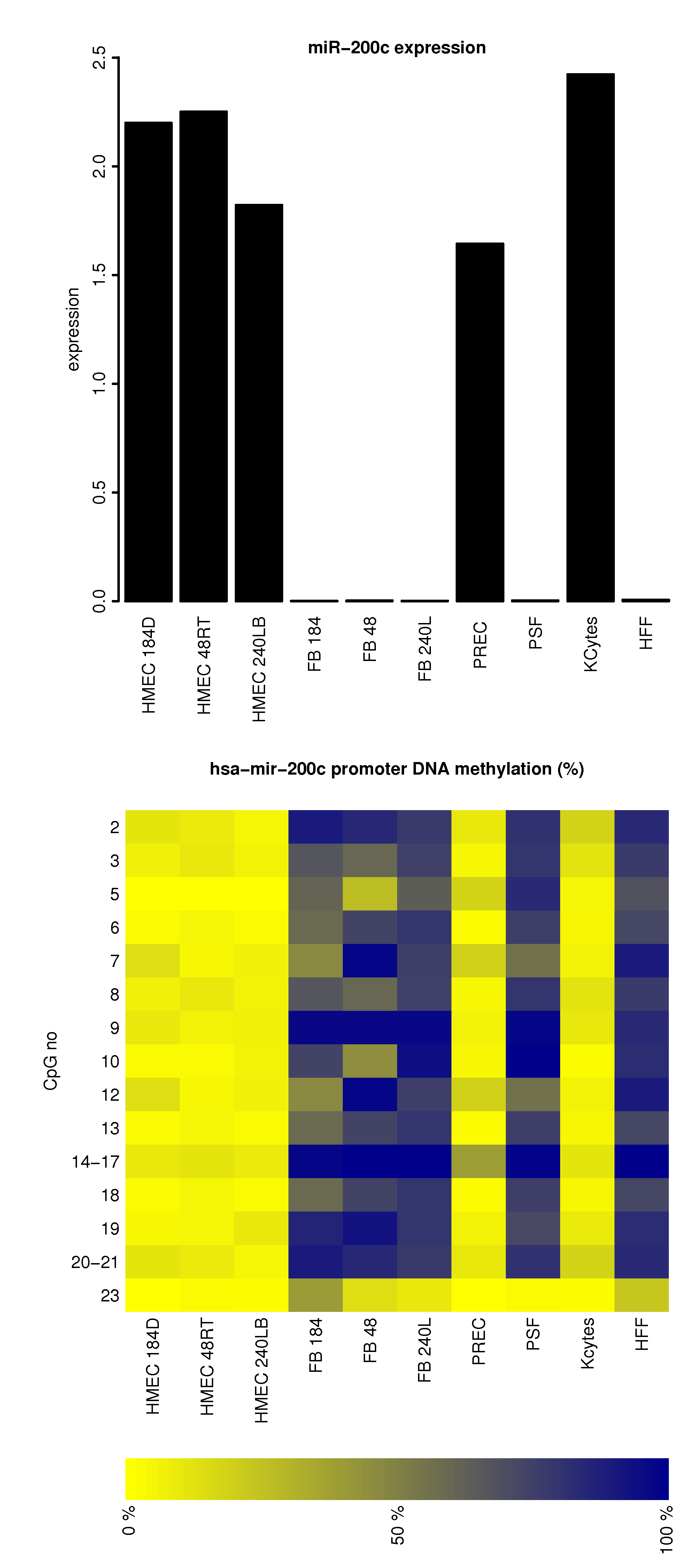

Supplement: Figure S2 — DNA methylation of the mir-200c CpG island inversely correlates with miR-200c expression in normal human samples. This figure summarizes data shown in Figure 1B and 2B. The upper panel shows the expression of miR-200c detected by real-time PCR. The bottom panel shows the methylation level of mir-200c CpG island region in the same human samples. The level of methylation of individual CpG units within the MassARRAY amplicon is displayed as a heatmap with the lowest methylation in yellow and the highest methylation in blue. The y-axis marks the individual CpG units. (0.19 MB TIF) [file pone.0008697.s002.tif]

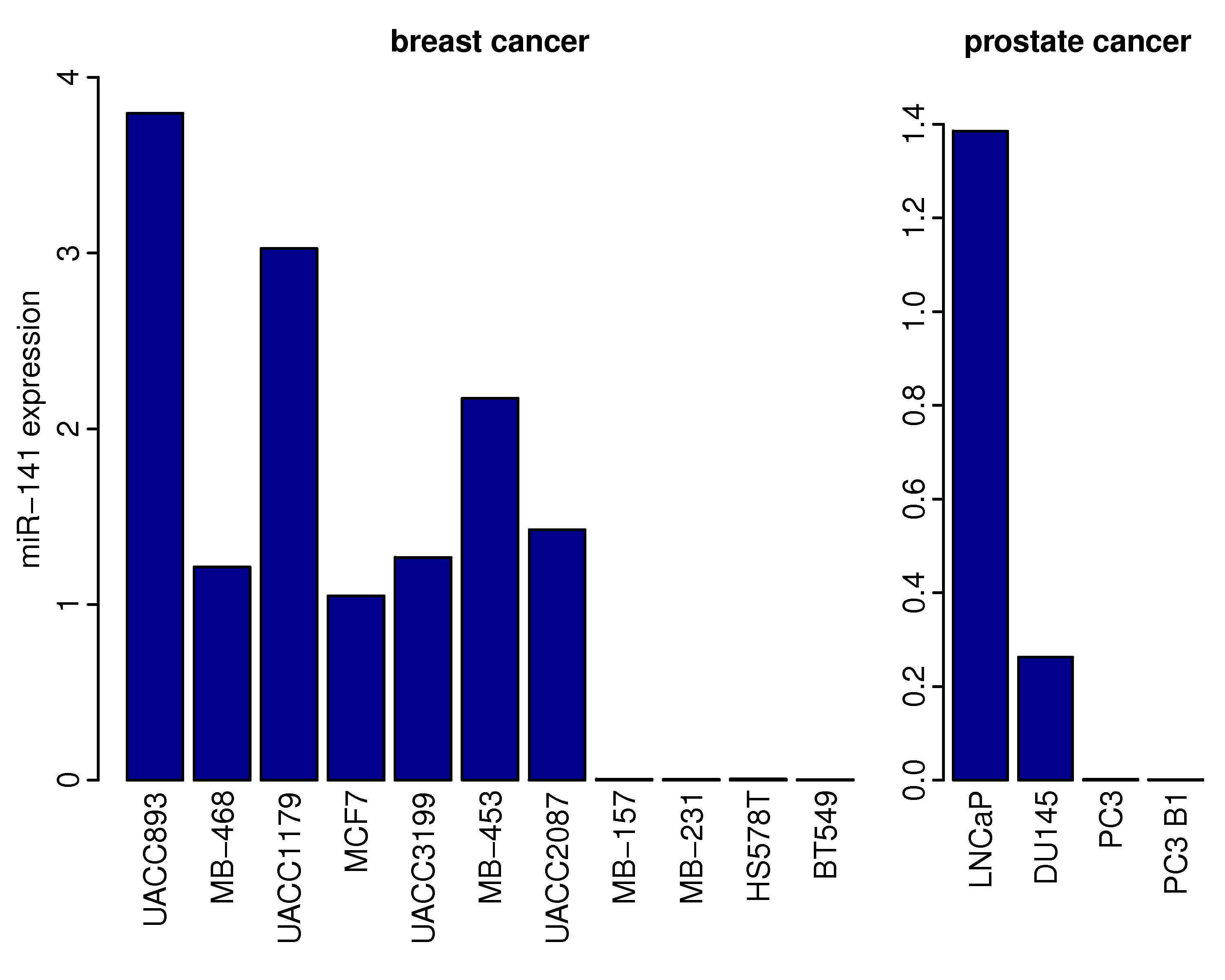

Supplement: Figure S3 — Real-time PCR assessment of miR-141 expression in breast and prostate cancer cell lines. The left panel shows the expression of miR-141 in eleven human breast cancer cell lines. The right panel shows the expression of miR-141 in four human prostate cancer cell lines. (0.14 MB TIF) [file pone.0008697.s003.tif]

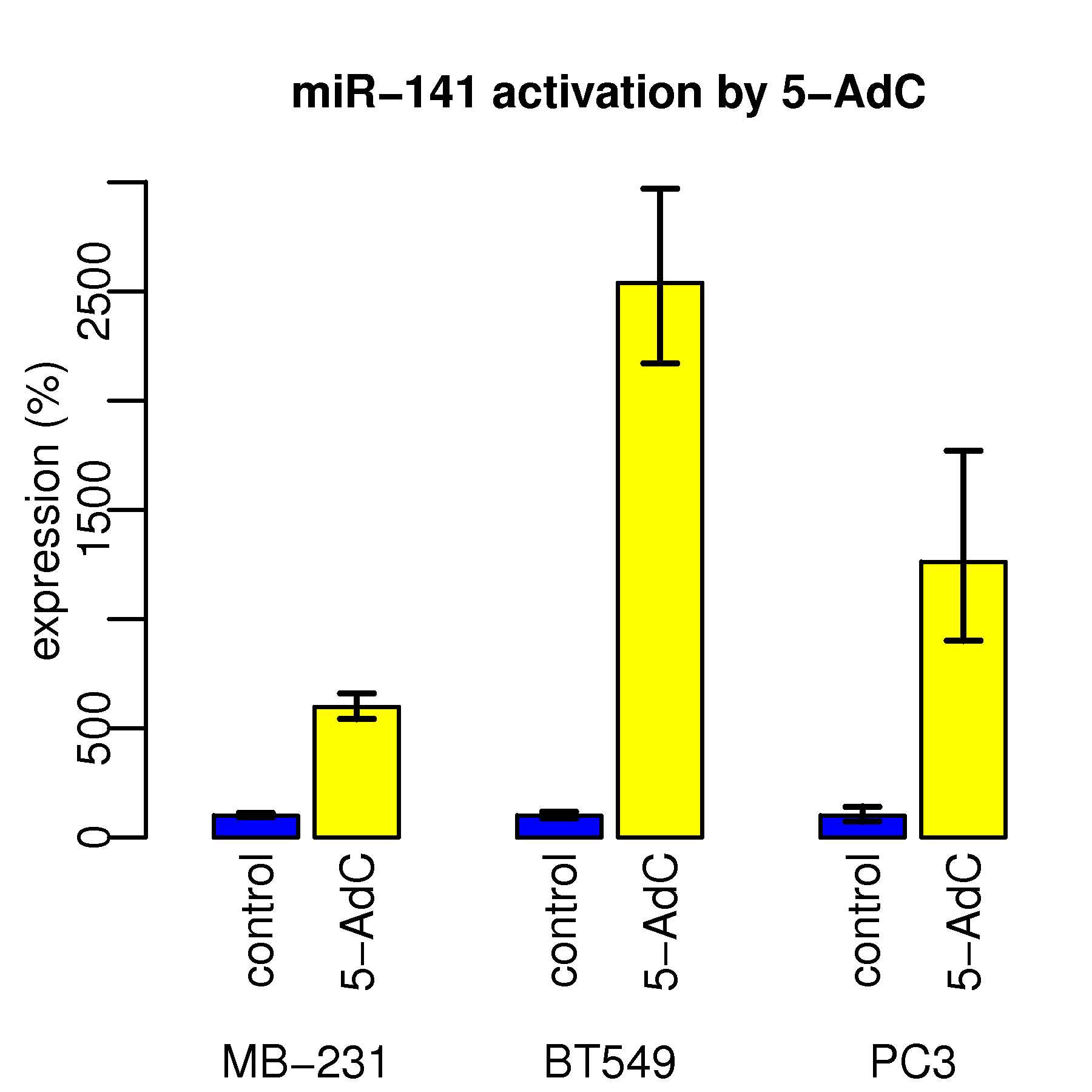

Supplement: Figure S4 — miR-141 expression in cancer cell lines is reactivated by 5-aza-2′-deoxycytidine treatment. Cells were treated with 3 µM 5-AdC for 96 h. The level of expression of miR-141 was measured by real-time PCR. The average of 4 measurements is displayed, the error bars show the standard error of measurement. The values were normalized to untreated controls (100%). (0.06 MB TIF) [file pone.0008697.s004.tif]

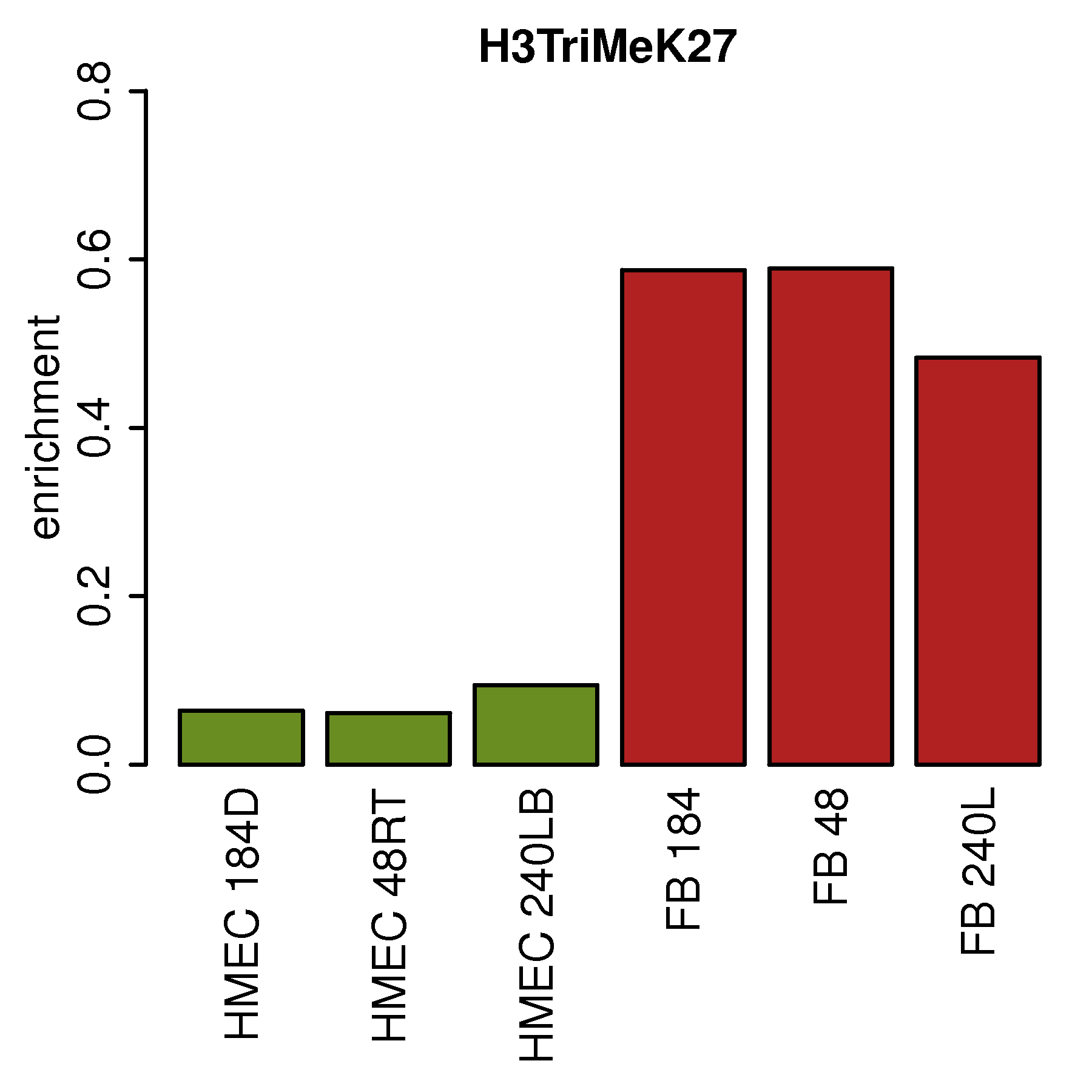

Supplement: Figure S5 — Histone H3 K27 trimethylation state of the mir-200c CpG island. Histone H3 lysine 27 trimethylation levels of the region of the mir-200c CpG island described in Figure 2A were analyzed by chromatin immunoprecipitation coupled to real-time PCR. Epithelial cells (HMEC) are shown in green and their isogenic fibroblasts (FB) are shown in red. The y-axis shows a lack of enrichment of the histone H3 K27 trimethylation mark within the mir-200c CpG island relative to input DNA in all the samples analyzed. (0.08 MB TIF) [file pone.0008697.s005.tif]
