# Supplementary material for: Role for DNA Methylation in the Regulation of miR-200c and miR-141 Expression in Normal and Cancer Cells
Source: PLoS One. 2010 Jan 13;5(1):e8697. doi: 10.1371/journal.pone.0008697 (PMC2805718; doi:10.1371/journal.pone.0008697)
Supplement: Table S1 — List of primer sequences used in the study (0.03 MB PDF) [file pone.0008697.s006.pdf]

Table S1

List of primer sequences used in the study

miRNA detection (miR-Q) primers:

miR-200c_RT	TGTCAGGCAACCGTATTCACCgtgagtggtTCCATCA
miR-200c_F	CGTCAGATGTCCGAGTAGAGGgggaacggcgTAATACTGCCGGGTAATG
miR-141_RT	TGTCAGGCAACCGTATTCACCgtgagtggtGCCATCT
miR-141_F	CGTCAGATGTCCGAGTAGAGGgggaacggcgTAACACTGTCTGGTAAAGA
let-7a_RT	TGTCAGGCAACCGTATTCACCgtgagtggtAACTATA
let-7a_F	CGTCAGATGTCCGAGTAGAGGgggaacggcgTGAGGTAGTAGGTTGTATA
Ampl_R	TGTCAGGCAACCGTATTCACC
Ampl_F	CGTCAGATGTCCGAGTAGAGGG

MassARRAY primers:

hsa-200c_10F	aggaagagagGTTGTAGTTAGTTAAGGGTTGGGGA
hsa-200c_T7R	cagtaatacgactcactatagggagaaggctCAACACCCACTCTCTAAAAACAAAT
mmu-200c_10F	aggaagagagGATGATATTGGGATAGGGGTTTTTA
mmu-200c_T7R	cagtaatacgactcactatagggagaaggctTTTCTTCTACACCACTCTAAAAATCAAA

ChIPs primers:

hsa-mir-200c promoter amplicon (universal probe #77):

200c_#77C_F	agggctcaccaggaagtgt
200c_#77C_R	agatccctggctcccatc
